# Bioelectrical impedance-derived total body water for predicting exertional rhabdomyolysis-induced acute kidney injury during military training: a prospective cohort study

**DOI:** 10.1186/s12882-026-05241-0

**Published:** 2026-08-01

**Authors:** Chaken Maniyan, Bancha Satirapoj, Ouppatham Supasyndh, Narongrit Siriwattanasit

**Affiliations:** Division of Nephrology, Department of Medicine, Phramongkutklao Hospital, College of Medicine, Bangkok, 10400 Thailand

**Keywords:** Exertional rhabdomyolysis, Acute kidney injury, Total body water, Bioelectrical impedance analysis, Body composition, Military recruits

## Abstract

**Background:**

Exertional rhabdomyolysis-induced acute kidney injury (AKI) is a serious complication among military recruits undergoing intensive physical training in hot climates. Current screening parameters, including body mass index and urine specific gravity, have limited predictive accuracy. We aimed to evaluate whether baseline body composition, particularly total body water (TBW) measured by dual-frequency bioelectrical impedance analysis (BIA), can predict exertional rhabdomyolysis-induced AKI, and to characterize dynamic body composition changes during intensive training in military recruits.

**Methods:**

A prospective cohort study was conducted among 301 healthy male conscripts from four military camps in Kanchanaburi, Thailand, during May–July 2017. Baseline body composition (TBW, muscle mass, and fat mass) was measured using a portable dual-frequency BIA device and reassessed biweekly throughout an 8-week intensive training program. The primary outcome was rhabdomyolysis-induced AKI, defined by clinical manifestations of rhabdomyolysis, creatine phosphokinase ≥ 5,000 U/L, and AKI according to KDIGO criteria. Logistic regression and receiver operating characteristic (ROC) analysis were performed to evaluate predictive performance.

**Results:**

Fifty-eight participants (19.3%) developed rhabdomyolysis-induced AKI. The AKI group had significantly lower baseline TBW than the non-AKI group (45.08 ± 2.95% vs. 54.31 ± 5.21%, *p* < 0.001) and demonstrated greater losses in muscle mass and fat mass during training. On multivariate logistic regression, lower baseline TBW was the strongest independent predictor (adjusted OR 1.56 per 1% decrease, 95% CI 1.26–1.92, *p* < 0.001), together with lower plasma potassium (adjusted OR 10.88 per 1 mEq/L decrease, 95% CI 1.87–63.16, *p* = 0.008) and lower exercise frequency (adjusted OR 12.80 per one fewer session/week, 95% CI 4.73–34.65, *p* < 0.001). Baseline TBW demonstrated an area under the ROC curve of 0.938 (95% CI 0.909–0.964). At a cut-off of TBW < 50%, sensitivity was 98.3%, specificity 74.9%, positive predictive value 48.3%, and negative predictive value 99.5%.

**Conclusion:**

Baseline TBW measured by portable BIA is a strong predictor of exertional rhabdomyolysis-induced AKI in military recruits undergoing intensive training. With a negative predictive value of 99.5% at TBW < 50%, this measure is particularly suited as a field-deployable rule-out screening tool. Multicenter validation is warranted before implementation in large-scale screening programs.

**Clinical trial registration:**

Not applicable.

## Introduction

Rhabdomyolysis is characterized by the breakdown of skeletal muscle cells, resulting in the release of intracellular contents — including myoglobin, creatine phosphokinase (CPK), electrolytes, and other sarcoplasmic proteins — into the systemic circulation [1, 2]. The most critical released component is myoglobin, an 18.8 kDa oxygen-carrying protein that, when filtered by the glomeruli in excessive amounts, can directly injure renal tubules and precipitate acute kidney injury (AKI) [3, 4]. AKI is the most serious complication of rhabdomyolysis, occurring in approximately 10–40% of affected patients [1, 5–7]. This complication carries substantial morbidity and mortality, particularly in settings where early recognition and intervention are delayed [8].

Beyond AKI, rhabdomyolysis may precipitate life-threatening electrolyte derangements — including hyperkalaemia, hyperphosphataemia and hypocalcaemia — as well as metabolic acidosis, compartment syndrome and, in severe cases, disseminated intravascular coagulation. AKI arises through three interrelated mechanisms: renal vasoconstriction and ischaemia from intravascular volume depletion; intratubular obstruction by myoglobin casts under acidic, low-flow conditions; and direct heme-mediated tubular cytotoxicity through oxidative injury and lipid peroxidation. Because each of these processes is amplified by hypovolaemia and low urinary flow, hydration status is a central and potentially modifiable determinant of whether rhabdomyolysis progresses to AKI.

Exertional rhabdomyolysis, the most common form of the condition, occurs predominantly during strenuous physical activity in hot and humid environments [9–11]. Military training represents a particularly high-risk setting, with reported incidence rates of 22 to 154 cases per 100,000 persons per year among armed forces personnel, and a 50% increase in cases observed in the U.S. military between 2010 and 2016 [12]. The highest rates are consistently reported among young male recruits and those in combat-specific training [12, 13]. In Thailand, approximately 60,000–80,000 young men are conscripted into the Royal Thai Army annually, undergoing a 10-week intensive basic training program [14]. A prospective study among Thai conscripts reported that BMI ≥ 30 kg/m² was independently associated with heat-related illnesses, with high rates of dehydration observed during basic military training across five regions in Thailand [14]. Monitoring of bedtime body temperature and body weight among recruits in Lopburi Province further demonstrated the feasibility of field-based surveillance in preventing heat stroke during training [15]. The tropical climate, particularly in regions such as Kanchanaburi Province — known for the highest heat index in the country — further amplifies this risk.

Established risk factors for exertional rhabdomyolysis include dehydration, low baseline physical fitness, prior heat injury, and physical exertion during warmer months [16, 17]. Dehydration, in particular, has been well documented to impair both physical and cognitive performance when body weight loss reaches 2% or more [18, 19]. Despite this knowledge, few studies have systematically evaluated field-deployable screening tools that can identify individuals at high risk for developing rhabdomyolysis-induced AKI prior to the onset of training [11, 20, 21]. Current conventional screening parameters — such as body mass index, urine specific gravity, and self-reported exercise history — offer limited predictive accuracy. There remains a critical need for a practical, objective, and easily measurable predictor that can be applied in austere field settings to enable targeted prevention.

Bioelectrical impedance analysis (BIA) is a noninvasive, rapid, and portable method for estimating body composition, including total body water (TBW), fat-free mass, and fat mass [22]. A National Institutes of Health technology assessment conference concluded that BIA provides a reliable and reasonable measure of TBW, with small intra- and interobserver variability [22]. Several military studies have evaluated BIA for body composition assessment, confirming its feasibility in field settings where conventional methods such as dual-energy X-ray absorptiometry or hydrostatic weighing are impractical [23–25]. Compared with other body composition measurement methods, BIA is relatively simple, does not require exposure to radioactivity or submersion in water, and can be performed rapidly in large groups — making it particularly well-suited for military screening applications [26, 27]. However, an important limitation must be acknowledged: TBW expressed as a percentage of body weight is inherently influenced by body fat mass, as adipose tissue contains relatively less water than lean tissue. Consequently, individuals with higher fat mass will have lower TBW% without necessarily being dehydrated. This distinction between low TBW% reflecting true hypohydration versus high adiposity must be considered when interpreting BIA-derived TBW as a hydration marker.

Given the high burden of exertional rhabdomyolysis-induced AKI among military recruits and the lack of a practical field-based screening tool, this study aimed to evaluate the predictive performance of baseline body composition — particularly TBW measured by portable dual-frequency BIA — for the development of rhabdomyolysis-induced AKI during intensive military training. As a secondary objective, we sought to characterize the dynamic changes in body composition (TBW, muscle mass, and fat mass) during the training period and to identify additional modifiable and non-modifiable risk factors associated with rhabdomyolysis-induced AKI in this population.

## Methods

### Study design and ethical approval

This was a prospective cohort study conducted in accordance with the Strengthening the Reporting of Observational Studies in Epidemiology (STROBE) guidelines [28]. The study protocol was reviewed and approved by the Institutional Review Board of the Royal Thai Army Medical Department. Written informed consent was obtained from all participants prior to enrollment.

### Setting and timeline

The study was conducted at four military-based training camps in Kanchanaburi Province, Thailand, between May and July 2017. This period corresponds to the early rainy season in Thailand, characterized by high ambient temperatures and humidity. Kanchanaburi Province is recognized as one of the regions with the highest heat index in Thailand, contributing to an elevated risk of heat-related illnesses among military personnel [14, 15].

The mean heat index during the training period was 51 °C, reflecting extreme heat stress conditions consistent with the highest risk category for heat-related illnesses.

### Participants

#### Eligibility criteria

Eligible participants were healthy male conscripts aged 18 years or older who were newly recruited into the Royal Thai Army for basic military training. Participants were required to be generally healthy, able to provide informed consent, and have no documented evidence of chronic disease affecting the neuromuscular, metabolic, or cutaneous systems, and no contraindication to bioelectrical impedance analysis.

#### Exclusion criteria

Participants were excluded if they had any of the following: significant volume depletion at baseline, hypertension, diabetes mellitus, chronic kidney disease, or any condition that would preclude body impedance analysis.

#### Recruitment and sampling

A total of 301 conscripts were enrolled from four military camps using probability proportional to size sampling to ensure representativeness across sites. All participants underwent an 8-week intensive military training program.

#### Data collection

All participants completed a standardized questionnaire at baseline, collecting information on current medication use, tobacco use, alcohol consumption (frequency per week in the month preceding recruitment), dietary habits, and physical training level prior to enlistment — including self-reported frequency of exercise (sessions per week), duration, and intensity. Additional data collected included educational level and gross annual income. Information regarding exercise schedule and medical symptoms in the days preceding any rhabdomyolysis event was also obtained.

#### Anthropometric measurements

Body weight was measured to the nearest 0.1 kg using a calibrated standard scale (Tanita TBF-543; Tanita Corporation, Tokyo, Japan) with participants wearing minimal clothing and bare feet. Body height was measured to the nearest 0.5 cm using a fixed wall-mounted stadiometer. Body mass index (BMI) was calculated as weight (kg) divided by height squared (m²). All anthropometric measurements were performed by a single trained investigator to minimize interobserver variability.

#### Body composition assessment by bioelectrical impedance analysis

Body composition was assessed using a portable dual-frequency bioelectrical impedance analysis (BIA) device (Tanita RD-953; Tanita Corporation, Tokyo, Japan). The device measures bioimpedance at two frequencies: 50 kHz and 6.25 kHz, with a current of 100 µA. Impedance values at 6.25 kHz were utilized for the estimation of total body water (TBW), while measurements at both frequencies were used for the estimation of muscle mass and fat mass.

Prior to each measurement, participants were instructed to remove all metallic objects from their clothing and body. Participants stood barefoot on the device platform with both feet in contact with the foot electrodes, maintaining a minimum distance of 10 cm between feet. Each measurement was performed in triplicate, and the mean value was recorded.

Baseline measurements were obtained prior to the commencement of training, and subsequent measurements were repeated at biweekly intervals (weeks 2, 4, 6, and 8) throughout the 8-week training program.

BIA has been established as a practical, noninvasive method for body composition assessment in field settings, with acceptable reliability and small intra- and interobserver variability [22, 23]. It has been validated for use in military populations where conventional methods such as dual-energy X-ray absorptiometry are impractical [24, 25].

All BIA measurements were performed in the fasting state at approximately 07:00 h, in an indoor setting with an ambient temperature of approximately 25 °C. Participants were instructed to void prior to measurement to minimize the influence of bladder volume on body water estimation.

Although the Tanita RD-953 has not been specifically validated in this study population, it utilizes the same dual-frequency impedance technology that has demonstrated acceptable accuracy for TBW estimation in comparable military and athletic cohorts [23, 24]. The absence of device-specific validation data in Thai military recruits is acknowledged as a limitation of this study.

### Laboratory methods

Baseline venous samples for CPK, serum creatinine, electrolytes and other biochemical parameters were obtained at enrolment, before the commencement of training. For outcome ascertainment, CPK and serum creatinine were measured at the time of clinical presentation and hospital admission in participants who developed clinical features of rhabdomyolysis. Serial serum creatinine was not measured in asymptomatic participants during the training period. Creatine phosphokinase (CPK) was measured by photometric assay using the Architect Creatine Kinase Reagent Kit (Abbott Laboratories, Abbott Park, IL, USA); the laboratory reference interval was 50–200 U/L for males above 17 years. Serum creatinine was measured by an enzymatic method using the Multigent Creatinine Assay (Sentinel CH, Milan, Italy) on an Abbott Architect c16000 analyzer; the laboratory reference interval was 0.5–1.0 mg/dL for males above 17 years. Estimated glomerular filtration rate (eGFR) was calculated using the Chronic Kidney Disease Epidemiology Collaboration (CKD-EPI) creatinine equation [29]. Serum electrolytes — including sodium, potassium, chloride, and bicarbonate — were measured using standard laboratory methods. Urine specific gravity was measured at baseline.

Serial serum creatinine monitoring was not performed in the non-AKI group during the training period. Consequently, subclinical or mild AKI episodes that did not result in clinical presentation and hospital admission may have been missed in this group, representing a potential source of outcome misclassification.

#### Outcome definition: exertional rhabdomyolysis-induced acute kidney injury

The primary outcome was the development of exertional rhabdomyolysis-induced AKI during the 8-week training period. A participant was classified as having the primary outcome if all of the following criteria were met upon hospital admission:

1. Clinical manifestations of rhabdomyolysis, defined as the presence of at least one of the following: urine color change to red or dark brown (assessed using the Royal Thai Army urine color chart), muscle pain, muscle cramp, muscle swelling, muscle stiffness, muscle tenderness, or skin induration or discoloration
2. Elevated creatine phosphokinase ≥ 5,000 U/L: A CPK threshold of ≥ 5,000 U/L (approximately 25 times the upper reference limit) was selected because the risk of rhabdomyolysis-induced AKI is low at lower enzyme concentrations and rises substantially above this level; ≥ 5,000 U/L is widely used to denote clinically significant rhabdomyolysis with meaningful nephrotoxic potential [3, 17]
3. Acute kidney injury, defined according to the Kidney Disease: Improving Global Outcomes (KDIGO) criteria [30] as any of the following: an increase in serum creatinine by ≥ 0.3 mg/dL (≥ 26.5 μmol/L) within 48 hours; an increase in serum creatinine to ≥ 1.5 times the baseline value, known or presumed to have occurred within the prior 7 days; or urine volume < 0.5 mL/kg/h for 6 hours (after volume repletion)
4. No active urinary sediment (absence of dysmorphic red blood cells and white blood cells < 10/μL), to exclude glomerulonephritis as a cause of AKI
5. No obstructive uropathy on renal ultrasonography

Participants who did not meet all five criteria were classified as the non-AKI group. It should be noted that the primary outcome required hospital admission for case ascertainment; subclinical AKI episodes that did not result in hospitalization may not have been captured. Additionally, serial serum creatinine monitoring was not performed in the non-AKI group during the training period; therefore, subclinical or mild AKI episodes in this group may have been missed, representing a potential source of outcome misclassification.

### Statistical analysis

Descriptive statistics were reported as means ± standard deviations (SD) for continuous variables and as percentages for categorical variables. Baseline characteristics between the AKI and non-AKI groups were compared using the independent samples t-test for continuous variables and the chi-square test or Fisher’s exact test for categorical variables. A p-value of less than 0.05 was considered statistically significant. The distribution of continuous variables was assessed using the Shapiro–Wilk test together with visual inspection of histograms and Q–Q plots. Normally distributed variables were compared using the independent-samples t-test and are presented as mean ± standard deviation; variables departing from normality were compared using the Mann–Whitney U test and are presented as median (interquartile range).

To identify independent risk factors for exertional rhabdomyolysis-induced AKI, a multiple logistic regression model was constructed using variables that demonstrated significant association with the outcome on univariate analysis. Candidate premorbid variables were screened by univariable logistic regression. Variables significant at *p* < 0.05 that were clinically relevant and statistically estimable were eligible for the multivariable model; variables exhibiting quasi-complete separation of the outcome (alcohol use, substance use, and weight loss ≥ 5%, for which one outcome cell was empty or near-empty) were excluded from regression and reported descriptively, because their odds ratios are not reliably estimable. The three eligible premorbid predictors — baseline TBW, plasma potassium, and exercise frequency — were entered as continuous variables (to avoid the separation and instability introduced by dichotomisation) into a multivariable logistic regression model adjusted for BMI and baseline CPK. This specification maintained an events-per-variable ratio of approximately 11.6 (58 events, 5 covariates), consistent with recommendations to limit overfitting. The reported AUC values represent apparent performance; internal validity of the combined model was additionally assessed by 10-fold cross-validation. Model calibration was assessed by the Hosmer–Lemeshow test. Model calibration was assessed by the Hosmer–Lemeshow test. Results were expressed as adjusted odds ratios (OR) with 95% confidence intervals (CI), adjusted for BMI and baseline CPK level.

Receiver operating characteristic (ROC) analysis was performed to evaluate the discriminative performance of baseline TBW and other candidate predictors for exertional rhabdomyolysis-induced AKI. The area under the ROC curve (AUC) was calculated using the trapezoidal rule as a global measure of discriminative ability, where a value of 1.0 indicates perfect discrimination and 0.5 indicates no discriminative ability. Sensitivity, specificity, positive predictive value (PPV), negative predictive value (NPV), and positive and negative likelihood ratios (LR+, LR-) were calculated at various cut-off points for baseline TBW to determine the optimal threshold for screening. ROC analysis was performed to assess the discriminative ability of baseline TBW, and the optimal screening cut-off was identified using the Youden index (J = sensitivity + specificity − 1). Combined prediction models incorporating multiple parameters were also evaluated to determine whether the addition of other variables to baseline TBW could improve predictive performance.

All statistical analyses were performed using STATA software version 13.0 (StataCorp LLC, College Station, TX, USA).

## Results

### Study population and baseline characteristics

A total of 301 male military recruits were enrolled and analyzed. The mean age was 22 ± 2.3 years. Fifty-eight participants (19.3%) developed exertional rhabdomyolysis-induced AKI during the 8-week training period (AKI group, *n* = 58), while 243 participants did not (non-AKI group, *n* = 243). The majority of AKI cases (65.5%) occurred within the first four weeks of training.

Baseline demographic, body composition, and laboratory characteristics are presented in Table 1. No significant differences were observed between the AKI and non-AKI groups in age (22 ± 2.5 vs. 21.9 ± 2.3 years, *p* = 0.9), educational level, annual income (61,487 ± 1,543 vs. 64,931 ± 988 baht, *p* = 0.78), body weight (71.66 ± 18.2 vs. 68.64 ± 12.2 kg, *p* = 0.233), BMI (26.32 ± 6.69 vs. 25.21 ± 4.5 kg/m², *p* = 0.233), baseline muscle mass (33.83 ± 1.62% vs. 33.89 ± 1.66%, *p* = 0.804), baseline fat mass (17.98 ± 2.7% vs. 18.1 ± 2.31%, *p* = 0.727), plasma sodium (142.56 ± 3.1 vs. 143.50 ± 2.1 mEq/L, *p* = 0.76), and plasma bicarbonate (27.40 ± 4.76 vs. 26.90 ± 5.82 mEq/L, *p* = 0.51).

**Table 1:** Baseline characteristics of AKI and non-AKI groups

Characteristic	Total (*N* = 301)	AKI (*n* = 58)	Non-AKI (*n* = 243)	*P*-value
Age (years)	22 *±* 2.3	22 *±* 2.5	21.9 *±* 2.3	0.900
Body weight (kg)	66.0 (57.6–77.4)	66.0 (57.8–86.8)	65.5 (57.9–75.7)	0.668
BMI (kg/m²)	24.3 (21.2–28.4)	24.3 (21.2–31.9)	24.1 (21.3–27.8)	0.668
Baseline TBW (%)	51.8 (48.0–57.4)	45.2 (43.0–47.4)	54.4 (50.0–58.3)	< 0.001
Baseline muscle mass (%)	34.2 (32.8–35.0)	35.0 (32.2–35.0)	34.2 (32.8–35.0)	0.820
Baseline fat mass (%)	17.8 (17.0–19.5)	17.5 (16.4–19.5)	17.8 (17.0–20.0)	0.206
Plasma potassium (mEq/L)	4.0 (3.6–4.3)	3.6 (3.5–4.0)	4.0 (3.6–4.3)	< 0.001
Plasma sodium (mEq/L)	141.5 ± 4.1	142.6 *±* 3.1	143.5 *±* 2.1	0.760
Plasma bicarbonate (mEq/L)	28.5 ± 3.1	27.4 *±* 4.8	26.9 *±* 5.8	0.510
Baseline CPK (U/L)	44.0 (34.0–65.0)	65.0 (36.2–85.8)	43.0 (34.0–65.0)	0.025
Urine specific gravity	1020 (1015–1020)	1015 (1015–1020)	1020 (1015–1020)	0.045
Exercise frequency (sessions/wk)	3.0 (2.0–4.0)	1.0 (1.0–2.0)	3.0 (3.0–4.0)	< 0.001
Weight loss, prior month (%)	3.3 (2.5–4.8)	6.0 (5.7–6.3)	3.0 (2.5–3.8)	< 0.001
Baseline creatinine (mg/dL)	1.09 ± 0.32	1.12 ± 0.21	1.07 ± 0.51	0.560
Baseline eGFR (mL/min/1.73 m²)	97.28 ± 4.38	97.19 ± 4.06	97.30 ± 0.51	0.868
Alcohol frequency (per week)	2.1 ± 1.7	2.7 *±* 0.7	1.4 *±* 0.7	< 0.001
Alcohol use n (%)	76 (25.2)	58 (100.0)	24 (9.9)	< 0.001

Abbreviations / notes: Continuous variables are median (IQR); all were non-normal by Shapiro–Wilk (*p* < 0.05), compared by Mann–Whitney U test. Categorical variables compared by Fisher’s exact test. AKI, acute kidney injury; BMI, body mass index; TBW, total body water; CPK, creatine phosphokinase; IQR, interquartile range. Age, sodium, bicarbonate, eGFR, Baseline creatinine, estimated glomerular filtration rate and alcohol frequency were normally distributed (Shapiro–Wilk *p* > 0.05) and are presented as mean ± SD

In contrast, several variables differed significantly between groups. The AKI group reported a lower frequency of exercise before recruitment (1.02 ± 0.13 vs. 3.49 ± 0.93 sessions/week, *p* < 0.001), more frequent alcohol consumption in the month preceding recruitment (2.7 ± 0.7 vs. 1.4 ± 0.7 times/week, *p* < 0.001), greater weight loss in the month before recruitment (4.50 ± 1.9% vs. 3.50 ± 2.4%, *p* = 0.001), lower plasma potassium (3.62 ± 0.33 vs. 4.00 ± 0.36 mEq/L, *p* = 0.001), and — most notably — significantly lower baseline total body water (45.08 ± 2.95% vs. 54.31 ± 5.21%, *p* < 0.001).

Notably, baseline fat mass percentage did not differ between the two groups (17.98 ± 2.7% vs. 18.1 ± 2.31%, *p* = 0.727), suggesting that the lower baseline TBW in the AKI group was not attributable to higher adiposity but rather reflected a genuinely lower hydration state.

### Body composition changes during intensive training

Longitudinal changes in body composition during the 8-week training period are presented in Table 2. Body weight decreased gradually in both groups throughout the training period, with no significant differences between the AKI and non-AKI groups at any time point (all *p* > 0.2).

**Table 2:** Body composition during the 8-week training period

Week	Total (*n* = 301)	AKI (*n* = 58)	Non-AKI (*n* = 243)	*P*-value
**Body weight (kg)**				
Baseline	69.22 ± 13.62	71.66 ± 18.20	68.64 ± 12.25	0.233
Week 2	67.63 ± 11.88	67.61 ± 16.76	67.63 ± 10.42	0.993
Week 4	67.81 ± 12.85	66.73 ± 17.71	68.06 ± 11.42	0.585
Week 6	68.17 ± 12.40	66.84 ± 16.96	68.49 ± 11.06	0.481
Week 8	67.25 ± 12.76	66.51 ± 16.30	67.43 ± 11.79	0.685
**Total body water (%)**				
Baseline	52.53 ± 6.07	45.08 ± 2.95	54.31 ± 5.21	< 0.001
Week 2	53.22 ± 5.77	47.64 ± 4.82	54.55 ± 5.16	< 0.001
Week 4	53.78 ± 5.60	46.97 ± 3.42	55.41 ± 4.73	< 0.001
Week 6	56.95 ± 5.62	57.48 ± 5.40	56.82 ± 5.67	0.428
Week 8	55.97 ± 4.58	55.87 ± 5.10	56.00 ± 4.46	0.851
**Muscle mass (%)**				
Baseline	33.88 ± 1.65	33.83 ± 1.62	33.89 ± 1.66	0.804
Week 2	29.52 ± 2.46	28.18 ± 2.92	29.83 ± 2.24	< 0.001
Week 4	27.82 ± 2.42	26.49 ± 2.88	28.14 ± 2.18	< 0.001
Week 6	26.13 ± 2.37	24.80 ± 2.84	26.44 ± 2.13	< 0.001
Week 8	24.43 ± 2.33	23.11 ± 2.81	24.75 ± 2.09	< 0.001
**Fat mass (%)**				
Baseline	18.08 ± 2.38	18.05 ± 2.30	18.08 ± 2.41	0.206
Week 2	15.75 ± 2.35	13.54 ± 1.72	16.28 ± 2.17	< 0.001
Week 4	14.84 ± 2.25	12.64 ± 1.61	15.37 ± 2.05	< 0.001
Week 6	13.77 ± 2.33	10.83 ± 1.38	14.47 ± 1.93	< 0.001
Week 8	12.86 ± 2.24	9.93 ± 1.26	13.56 ± 1.81	< 0.001

Abbreviations / notes: Values are mean ± SD; compared by Mann–Whitney U test (all distributions non-normal). Note the paradoxical week-2 trajectories (TBW rises while muscle and fat fall in the AKI group) are interpreted as impedance artefact during evolving AKI — see Discussion. AKI, acute kidney injury; SD, standard deviation

Total body water showed a markedly different trajectory between groups. The AKI group, which started with a significantly lower baseline TBW (45.08 ± 2.95% vs. 54.31 ± 5.21%, *p* < 0.001), demonstrated a progressive increase in TBW over the training period. This difference remained statistically significant at week 2 (47.64 ± 4.82% vs. 54.55 ± 5.16%, *p* < 0.001) and week 4 (46.97 ± 3.42% vs. 55.41 ± 4.73%, *p* < 0.001), but converged by week 6 (57.48 ± 5.4% vs. 56.82 ± 5.67%, *p* = 0.428) and week 8 (55.87 ± 5.1% vs. 56 ± 4.46%, *p* = 0.851).

Muscle mass declined in both groups, but the AKI group demonstrated significantly greater losses than the non-AKI group from week 2 onward: week 2 (28.18 ± 2.92% vs. 29.83 ± 2.24%, mean difference − 1.65%, *p* < 0.001), week 4 (26.49 ± 2.88% vs. 28.14 ± 2.18%, *p* < 0.001), week 6 (24.8 ± 2.84% vs. 26.44 ± 2.13%, *p* < 0.001), and week 8 (23.11 ± 2.81% vs. 24.75 ± 2.09%, *p* < 0.001).

Fat mass also declined in both groups, with significantly greater losses in the AKI group emerging from week 2: week 2 (14.99 ± 2.7% vs. 15.93 ± 2.23%, mean difference − 0.95%, *p* = 0.006), week 4 (14.09 ± 2.58% vs. 15.03 ± 2.12%, *p* = 0.004), and week 6 (12.79 ± 2.72% vs. 14 ± 2.17%, *p* = 0.002).

### Risk factors for exertional rhabdomyolysis-induced AKI

Univariate and multivariate logistic regression analyses are presented in Table 3. On multivariate analysis, adjusted for BMI and baseline CPK level, the following variables were independently associated with the development of exertional rhabdomyolysis-induced AKI:

**Table 3:** Logistic regression for rhabdomyolysis-induced AKI (continuous predictors)

Predictor	Crude OR (95% CI)	*p*	Adjusted OR (95% CI)	*p*
Baseline TBW, per 1% lower	1.72 (1.49–1.99)	< 0.001	1.56 (1.26–1.92)	< 0.001
Plasma potassium, per 1 mEq/L lower	8.73 (3.67–20.76)	< 0.001	10.88 (1.87–63.16)	0.008
Exercise frequency, per 1 session/wk fewer	11.25 (6.33–20.01)	< 0.001	12.80 (4.73–34.65)	< 0.001
BMI, per 1 kg/m²	—	—	1.06 (0.95–1.19)	0.286
Baseline CPK, per 1 U/L	—	—	1.00 (0.98–1.02)	0.776

Abbreviations / notes: Multivariable model adjusted for BMI and baseline CPK. Odds ratios for TBW, potassium and exercise frequency are expressed per 1-unit decrement so that OR > 1 denotes higher risk. Model calibration: Hosmer–Lemeshow χ²=2.35, *p* = 0.968. OR, odds ratio; CI, confidence interval; TBW, total body water; BMI, body mass index; CPK, creatine phosphokinase

- Lower baseline TBW was the strongest independent predictor (adjusted OR 1.56 per 1% decrease, 95% CI 1.26–1.92, *p* < 0.001).
- Lower plasma potassium (adjusted OR 10.88 per 1 mEq/L decrease, 95% CI 1.87–63.16, *p* = 0.008).
- Lower exercise frequency (adjusted OR 12.80 per one fewer session/week, 95% CI 4.73–34.65, *p* < 0.001).

### Predictive performance of baseline TBW and other parameters

The discriminative performance of individual and combined parameters for predicting exertional rhabdomyolysis-induced AKI is presented in Table 4.

**Table 4:** Performance of single and combined parameters for predicting exertional rhabdomyolysis-induced AKI

Predictor	AUC (95% CI)
Baseline TBW	0.938 (0.909–0.964)
Exercise frequency	0.943 (0.917–0.964)
Plasma potassium	0.716 (0.638–0.786)
Urine specific gravity	0.581 (0.494–0.666)
BMI	0.518 (0.418–0.615)
Combined premorbid model (TBW + potassium + exercise frequency)	0.981 (0.967–0.992)

Abbreviations / notes: AUC from univariable logistic models (combined model from predicted probabilities of the multivariable model); 95% CI by bootstrap (1000 replicates). AUC, area under the ROC curve; TBW, total body water; BMI, body mass index

Among single parameters, baseline TBW and exercise frequency showed the highest discrimination (AUC 0.938, 95% CI 0.909–0.964; and 0.943, 95% CI 0.917–0.964), followed by plasma potassium (0.716), urine specific gravity (0.581), and BMI (0.518). Combined prediction models demonstrated further improvement in discriminative ability. A combined premorbid model incorporating baseline TBW, plasma potassium, and exercise frequency achieved an AUC of 0.981 (95% CI 0.967–0.992). On 10-fold cross-validation, the combined model retained an AUC of 0.978 (apparent AUC 0.981; optimism 0.003), indicating minimal overfitting.

### Optimal cut-off for baseline TBW

**Fig. 1:**
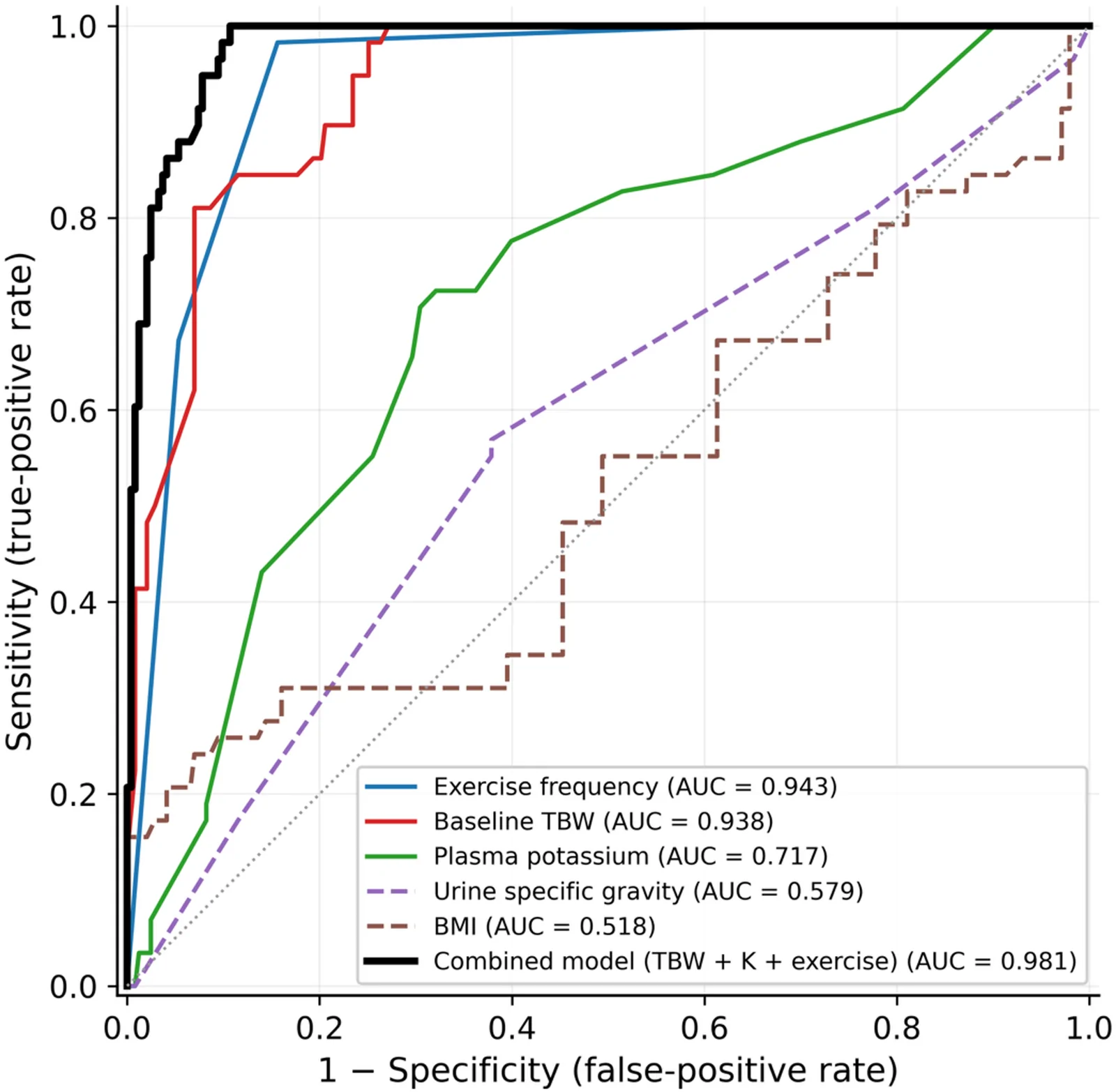
ROC curves for prediction of rhabdomyolysis-induced AKI. Abbreviations: BMI, body mass index; TBW, total body water; K, plasma potassium

Sensitivity, specificity, predictive values, and likelihood ratios at various baseline TBW cut-off points are presented in Table 5. and Fig. 1.

**Table 5:** Operating characteristics of baseline TBW at various cut-offs for predicting exertional rhabdomyolysis-induced AKI

TBW cut-off	Sn (%)	Sp (%)	PPV (%)	NPV (%)	LR+	LR−	Accuracy (%)	Youden
< 44%	41.4	97.9	82.8	87.5	20.11	0.60	87.0	0.393
< 45%	50.0	97.1	80.6	89.1	17.36	0.51	88.0	0.471
< 47%	69.0	93.0	70.2	92.6	9.86	0.33	88.4	0.620
< 48%	81.0	91.4	69.1	95.3	9.38	0.21	89.4	0.724
< 49%	84.5	84.0	55.7	95.8	5.26	0.18	84.1	0.684
< 50%	98.3	74.9	48.3	99.5	3.91	0.02	79.4	0.732
< 51%	100.0	66.7	41.7	100.0	3.00	0.00	73.1	0.667
< 52%	100.0	61.3	38.2	100.0	2.59	0.00	68.8	0.613
< 55%	100.0	49.0	31.9	100.0	1.96	0.00	58.8	0.490

Abbreviations / notes: Optimal cut-off by the Youden index (J = sensitivity + specificity − 1) = TBW < 50% (J=0.732). Area under the ROC curve for baseline TBW = 0.938 (95% CI 0.909–0.964). Sn, sensitivity; Sp, specificity; PPV/NPV, positive/negative predictive value; LR+/LR−, positive/negative likelihood ratio; TBW, total body water

At a cut-off of TBW < 50%, the test characteristics were as follows: sensitivity 98.3%, specificity 74.9%, positive predictive value (PPV) 48.3%, negative predictive value (NPV) 99.5%, positive likelihood ratio (LR+) 3.91, negative likelihood ratio (LR-) 0.02, and overall accuracy 79.4%.

Additional cut-off points demonstrated the expected trade-off between sensitivity and specificity. The cut-off of TBW < 50% was selected as optimal, balancing high sensitivity with acceptable specificity. This threshold demonstrated particular strength as a rule-out screening tool: among recruits with baseline TBW ≥ 50%, the probability of subsequently developing rhabdomyolysis-induced AKI was only 0.5% (NPV 99.5%). Conversely, the PPV of 48.3% indicates that approximately half of those flagged as high-risk at this cut-off would not develop AKI, which must be considered when implementing this threshold in screening programs for large populations.

## Discussion

This prospective cohort study demonstrated that baseline total body water (TBW) measured by portable dual-frequency bioelectrical impedance analysis (BIA) was the strongest predictor of exertional rhabdomyolysis-induced AKI among Thai military recruits undergoing intensive training in a tropical environment. With an area under the ROC curve of 0.938, baseline TBW outperformed conventional screening parameters such as BMI (AUC 0.518) and urine specific gravity (AUC 0.581). At a cut-off of TBW < 50%, the test demonstrated high sensitivity (98.3%) and an exceptionally high negative predictive value (99.5%), making it well-suited as a field-deployable rule-out screening tool. Additionally, dynamic changes in body composition during the first two weeks of training — including increased TBW, decreased muscle mass, and decreased fat mass — were independently associated with AKI development.

The observed incidence of rhabdomyolysis-induced AKI in this study was 19.3% (58 of 301 participants), which is substantially higher than the incidence rates of 22 to 154 cases per 100,000 persons per year reported from U.S. military surveillance data [12]. Several factors likely account for this discrepancy. First, this study employed active biweekly surveillance with systematic clinical assessment and laboratory evaluation of all participants who developed clinical signs of rhabdomyolysis, which would capture cases that might go undetected under passive surveillance systems. Second, the study was conducted in Kanchanaburi Province during the hot season (May–July), with a mean heat index of 51 °C — representing extreme heat stress conditions that substantially amplify the risk of exertional rhabdomyolysis [14]. Third, unlike population-based incidence rates that include all military personnel, this study focused exclusively on a high-risk cohort of new recruits undergoing the most physically demanding phase of their military service. Finally, the U.S. incidence figures are typically based on hospitalization or medical encounter data, whereas this study used a lower CPK threshold (≥ 5,000 U/L) combined with KDIGO AKI criteria applied through active case ascertainment, which may have captured additional cases. These differences in study design, case definition, and environmental context should be considered when comparing incidence rates across studies, and the 19.3% figure should be interpreted as the incidence within this specific enriched high-risk cohort rather than as a population-based incidence rate.

The finding that low baseline TBW was the strongest predictor of rhabdomyolysis-induced AKI is biologically plausible. Adequate hydration is critical for maintaining renal perfusion, diluting nephrotoxic myoglobin within the tubular lumen, and promoting urinary flow to prevent myoglobin cast formation [3, 16]. Individuals entering intensive training in a dehydrated state may have diminished renal reserve, predisposing them to AKI when exposed to the nephrotoxic insult of myoglobin.

An important methodological consideration, however, is that TBW expressed as a percentage of body weight is inherently influenced by body composition — specifically, adipose tissue contains proportionally less water (~ 10%) than lean tissue (~ 73%). Therefore, individuals with higher body fat would exhibit lower TBW% even if their absolute hydration status is adequate. If this confounding were present, the observed association between low TBW% and AKI could reflect higher adiposity rather than true dehydration.

Importantly, our data provide evidence against this alternative explanation. Baseline fat mass percentage did not differ significantly between the AKI and non-AKI groups (17.98 ± 2.7% vs. 18.1 ± 2.31%, *p* = 0.727), nor did BMI (26.32 ± 6.69 vs. 25.21 ± 4.5 kg/m², *p* = 0.233). This suggests that the lower baseline TBW in the AKI group was not driven by higher adiposity but rather reflected a genuinely lower hydration state. Nevertheless, we acknowledge that BIA-derived TBW% cannot fully disentangle the contributions of hydration and body fat, and future studies incorporating direct hydration markers (such as plasma osmolality or urine osmolality) alongside BIA would be valuable for clarifying this relationship.

The dynamic body composition changes observed during training are consistent with the pathophysiology of rhabdomyolysis-induced AKI. The progressive increase in TBW within the AKI group during the oliguric phase of AKI is consistent with the findings of Bluemle et al. (1956) [31], who demonstrated solute retention and increased total body water measured by deuterium oxide dilution during oliguric AKI. The concurrent loss of muscle mass and fat mass in the AKI group likely reflects the catabolic state associated with systemic inflammation. Rhabdomyolysis triggers the release of immunostimulatory molecules from damaged muscle cells, which activate inflammatory pathways including the NLRP3 inflammasome [32] and the production of pro-inflammatory cytokines such as TNF-α [33] and TGF-β, driving proteolysis and lipolysis [4].

An important interpretive consideration is the temporal relationship between early body composition changes and AKI development. Although the week-2 dynamic changes — TBW increase, muscle-mass decline and fat-mass decline — were strongly associated with AKI on univariable analysis, they discriminated the outcome almost perfectly (area under the ROC curve ≈ 1.00), and every recruit who developed AKI showed such changes. Given that 65.5% of AKI cases occurred within the first four weeks of training, this degree of separation indicates that these changes largely represent early consequences of an already-initiated rhabdomyolysis–AKI process rather than antecedent risk factors: the week-2 TBW increase plausibly reflects early fluid retention during the oliguric phase of evolving AKI, and the muscle-mass decline reflects ongoing muscle breakdown. We therefore excluded the week-2 variables from the prediction model and regard them as early markers of evolving disease — useful for early detection and monitoring — rather than as pre-morbid predictors. In contrast, baseline TBW, measured before training onset, is free from this reverse-causation concern, strengthening its validity as a true pre-morbid predictor.

The clinical utility of baseline TBW as a screening tool warrants careful consideration of both its strengths and limitations. At the optimal cut-off of TBW < 50%, the NPV of 99.5% indicates that recruits with baseline TBW ≥ 50% have an extremely low probability of developing rhabdomyolysis-induced AKI. This makes the test particularly valuable as a rule-out tool: in a population of 100,000 Thai military recruits, those above the threshold can be reassured and proceed with standard training protocols with a high degree of confidence.

However, the PPV of 48.3% means that more than half of recruits flagged as high-risk at this threshold would not develop AKI. In a hypothetical screening program for 100,000 recruits, a substantial number of individuals would be unnecessarily flagged, potentially leading to resource allocation challenges, training modifications without clinical benefit, or psychological impact from being labeled as high-risk. The trade-off between sensitivity and PPV is inherent to any screening test applied in a population with moderate disease prevalence [11], and the acceptable balance depends on the consequences of missed cases (false negatives) versus unnecessary interventions (false positives).

We propose that the TBW < 50% threshold is most appropriately used as a first-line screening tool to identify a low-risk group that requires no additional intervention (TBW ≥ 50%), while those below the threshold undergo enhanced surveillance — including more frequent clinical assessment, hydration optimization, and serial laboratory monitoring during the early weeks of training — rather than exclusion from training. Because low baseline exercise frequency was itself an independent predictor (adjusted OR 12.80 per one fewer session/week), the elevated risk in less-conditioned recruits is plausibly mediated by abrupt exposure to intense exertion; a graded, progressive increase in training load for this subgroup is therefore a logical, low-cost preventive measure that complements hydration optimisation. The combined model incorporating baseline TBW, serum potassium, and exercise frequency (AUC 0.981) may further refine risk stratification; however, external validation in an independent cohort is required before clinical implementation.

This study has several notable strengths. To our knowledge, this is among the first prospective studies to evaluate BIA-derived body composition parameters as predictors of exertional rhabdomyolysis-induced AKI. The longitudinal design with biweekly body composition measurements provided a detailed characterization of dynamic changes throughout the training period. The study was conducted in a real-world military setting with standardized training protocols, enhancing the ecological validity of the findings. BIA is a practical, noninvasive, and field-deployable tool that can be performed rapidly in large groups — characteristics essential for any screening instrument intended for military application [22, 23]. Furthermore, the use of a well-defined composite outcome incorporating clinical criteria, CPK thresholds, and KDIGO AKI criteria strengthened case ascertainment.

Several limitations of this study should be acknowledged. First, the study was conducted in a single geographic area (Kanchanaburi Province) during the hot season, with a mean heat index of 51 °C. Results may not be generalizable to settings with different climatic conditions or training intensities. Second, the study enrolled only male conscripts; the applicability to female military personnel or voluntary soldiers is unknown. Third, The outcome definition has two related limitations. First, requiring clinical manifestations and a composite definition (rather than KDIGO serum-creatinine/urine-output criteria alone) identified only clinically significant, myoglobinuric AKI and underestimated the total burden of AKI, including subclinical episodes; this composite was chosen deliberately to capture the phenotype attributable to exertional rhabdomyolysis and to avoid misclassifying transient prerenal or heat-related creatinine elevations. Second, serial creatinine was not measured in the non-AKI group, so subclinical AKI in that group may have been missed. Because any such misclassification would bias the group comparison toward the null, the associations reported here are likely to be conservative. Future studies should apply KDIGO criteria with protocolised serial creatinine in all participants. Forth, the BIA device used (Tanita RD-953) is a consumer-grade instrument that has not been specifically validated against reference methods in this study population; although dual-frequency BIA technology has demonstrated acceptable accuracy in comparable military cohorts [23, 24], device-specific validation data are lacking. Fifth, the multivariate model was adjusted for BMI and baseline CPK, but did not include potentially important confounders such as ambient temperature and humidity on specific training days, individual fluid intake during training, or training intensity — variables that were not systematically collected but could influence AKI risk. Sixth, no a priori sample size calculation was performed, and multiple prediction models were evaluated (Table 4) without formal adjustment for multiple comparisons, which may increase the risk of false-positive findings. Seventh, the device returned only composition outputs; raw resistance, reactance, phase angle and separate extracellular/intracellular water were unavailable, precluding bioelectrical impedance vector analysis (BIVA) or calculation of the ECW/TBW ratio that would characterise these changes directly. Finally, the combined prediction model achieving an AUC of 0.981 was developed and evaluated in the same cohort without external validation. Such near-perfect discrimination in a single-center study with 58 events likely reflects some degree of overfitting, and the model’s performance should be confirmed in an independent cohort before it is used for clinical decision-making.

## Conclusion

Baseline total body water measured by portable dual-frequency bioelectrical impedance analysis was the strongest predictor of exertional rhabdomyolysis-induced acute kidney injury among Thai military recruits undergoing intensive training in a tropical environment. At a cut-off of TBW < 50%, this measure demonstrated high sensitivity (98.3%) and an exceptionally high negative predictive value (99.5%), making it particularly suited as a field-deployable rule-out screening tool to identify low-risk individuals prior to training. Additional independent risk factors included low plasma potassium and low baseline exercise frequency. These findings support the integration of BIA-based TBW screening into military pre-training health assessments to enable targeted prevention strategies, including hydration optimization and enhanced surveillance for recruits identified as high-risk. In addition to hydration optimisation and enhanced surveillance for high-risk recruits, these findings underscore the importance of a gradual, progressive increase in training intensity — particularly for less-conditioned individuals and those reporting low baseline exercise frequency — to reduce the risk of exertional rhabdomyolysis-induced AKI. Multicenter validation in diverse military populations and climatic settings is warranted to confirm the generalizability and clinical utility of these findings before large-scale implementation.

## Data Availability

The datasets generated and/or analyzed during the current study are available from the corresponding author, Narongrit Siriwattanasit, upon reasonable request (Email: nsiriwattanasit@gmail.com).
